# Supplementary material for: Higher Radiation Dose to the Immune Cells Correlates with Worse Tumor Control and Overall Survival in Patients with Stage III NSCLC: A Secondary Analysis of RTOG0617
Source: Cancers (Basel). 2021 Dec 8;13(24):6193. doi: 10.3390/cancers13246193 (PMC8699524; doi:10.3390/cancers13246193)
Supplement: Supplementary file 1 [file cancers-13-06193-s001.zip › cancers-1288194-supplementary.pdf]

Table S1. Patient characteristics and their effects on survival.

Characteristics	Dose <67 Gy (*n* = 285)	Dose ≥67 Gy (*n* = 171)	Total (*n* = 456)	*p*
Age (years)	64 (37–82)	64 (41–84)	64 (37–84)	0.89
Gender				0.43
Male	174 (61%)	98 (57%)	272 (60%)	
Female	111(39%)	73 (435)	184 (40%)	
Race				0.44
White	242 (85%)	152 (89%)	394 (86%)	
Black	31 (11%)	13 (8%)	44 (10%)	
Others	12 (4%)	6 (3%)	18 (4%)	
Zubrod status				0.90
0	170 (60%)	101 (59%)	271 (59%)	
1	115 (40%)	70 (41%)	185 (41%)	
Histology				0.68
Squamous	122 (43%)	73 (43%)	195 (43%)	
Adeno	114 (40%)	65 (38%)	179 (39%)	
Others	48 (17%)	33 (19%)	81 (18%)	
AJCC stage				0.73
IIIa	190 (67%)	111 (65%)	301 (66%)	
IIIb	94 (33%)	59 (35%)	153 (34%)	
RT technique				0.48
3D-CRT	153 (54%)	86 (50%)	239 (52%)	
IMRT	132 (46%)	85 (50%)	217 (48%)	
PET staging				0.37
No	23 (8%)	18 (11%)	41 (9%)	
Yes	262 (92%)	153 (90%)	415 (91%)	
Tumor location				0.48
LLL/central	32 (11%)	23 (14%)	55 (12%)	
Others	253 (89%)	148 (87%)	401 (88%)	
Weight loss/month	0 (0–9%)	0 (0–7%)	0 (0–9%)	0.99
Esophagitis grade				0.03
Grade <3	253 (89%)	139 (81%)	392 (86%)	
Grade ≥3	32 (11%)	32 (19%)	64 (14%)	
Received full chemo				0.095
No	42 (15%)	16 (9%)	58 (13%)	
Yes	243 (85%)	155 (91%)	398 (87%)	
GTV (cc)	92.7 (4.6–960.7)	93.7 (5.4–698.9)	92.7 (4.6–961)	0.48
Mean lung dose (Gy)	17.4 (5.4–31.7)	20.1 (5.1–32.7)	18.4 (5.1–32.7)	<0.0001
Mean heart dose (Gy)	13.2 (0–47.1)	12.6 (0.4–49.4)	12.7 (0–49.4)	0.45
Integral total body dose (Gy•L)	206 (62–545)	244 (104–464)	218 (62–545)	<0.0001
EDIC (Gy)	5.58 (2.05–12.20)	6.34 (2.14–11.59)	5.94 (2.05–12.2)	<0.0001

Abbreviations: RT, Radiotherapy; 3D-CRT, 3-D conformal radiation therapy; IMRT, intensity modulated radiation therapy; LLL, low left lobe; GTV, gross tumor volume; EDIC, effective dose to immune cells; OS, overall survival; PFS, progression-free survival; LPFS, local progression-free survival.
